# Practical Gynecology

**Published:** 1878-06

**Authors:** 


### Books Reviewed.

*Practical Gynecology*—A Hand-Book of the Diseases of Women. By Heywood Smith, M. A., M. D. Oxon., member of the Royal College of Physicians, etc.; with illustrations. Philadelphia: Lindsay & Blackiston. 1878. Buffalo: T. H. Butler. pp. 205. Price, $2.00.

This is the second volume of the Student's Guide series. The author in his introductory statement says that "In the female, as in the vegetable kingdom, the organs of reproduction constitute the whole essence of the creation." Having passed the introduction, we find a book written with studied brevity and with evidence of some knowledge of diseases of women. The first chapter on Means of Diagnosis, occupies twenty-one pages, while the next, on Syphilis, Cancer and Phthisis has four, though Cancer is again briefly discussed.

The consideration of local diseases is commenced with the ovaries, and follows the anatomy of the parts from above downwards, and from within outwards. Afterwards come diseases of the Mamma, Functional Diseases, Diseases connected with Pregnancy, with Parturition, and consequent on Parturition, as subjects of chapters, and in the order named. An appendix of remedies and index close the volume.

We should prefer to give for guides to American students books by American authors. An edition of English authors upon different subjects is very well to use for comparison, but we hope it will in no case supercede or be used in place of works of equal or superior merit by American physicians, than whom we believe there are none more discreet, conscientious or capable in the world.

